# Prevalence and penetrance of *BRCA1* and *BRCA2* mutations in a population-based series of breast cancer cases

**DOI:** 10.1054/bjoc.2000.1407

**Published:** 2000-10-26

**Authors:** Anglian Breast Cancer Study Group

**Affiliations:** See Appendix 2 for contributors

**Keywords:** breast, ovarian, cancer, BRCA1, BRCA2, prevalence, penetrance

## Abstract

Estimates of the contribution of *BRCA1* and *BRCA2* to breast cancer incidence in outbred populations have been based on studies that are either small or have selected for cases diagnosed at an early age. Only one of these has reported an estimate of the breast cancer risk associated with a mutation in these genes, and there is no published ovarian cancer risk estimate derived from a population-based case series. We screened a population-based series of breast cancer cases diagnosed before the age of 55 for mutations in *BRCA1* and *BRCA2*. Pedigree information from the mutation carriers was used to estimate penetrance and the proportion of familial risk of breast cancer due to *BRCA1* and *BRCA2*. We identified eight (0.7%)*BRCA1* and 16 (1.3%)*BRCA2* mutation carriers in 1220 breast cancer cases (actual sample size 1435 adjusted for 15% polymerase chain reaction failure rate). Mutation prevalence was substantially higher in cases diagnosed before 35 years-of-age and with increasing number of relatives affected with breast or ovarian cancer. However, most mutation carriers were diagnosed in the older age groups, and a minority reported a first-degree relative with breast cancer. Breast cancer penetrance by age 80 was estimated to be 48% (95% CI 7–82%) for *BRCA1* mutation carriers and 74% (7–94%) for *BRCA2* mutation carriers. Ovarian cancer penetrance for *BRCA1* and *BRCA2* combined was 22% (6–65%) by age 80. 17% of the familial risk of breast cancer was attributable to *BRCA1* and *BRCA2*. At birth, the estimated prevalence of *BRCA1* mutation carriers was 0.07% or 0.09% depending on the penetrance function used for the calculation. For *BRCA2* the birth prevalence estimates were 0.14% and 0.22%. Mutations in the genes *BRCA1* and *BRCA2* are rare in the population and account for a small fraction of all breast cancer in the UK. They account for less than one fifth of the familial risk of breast cancer. Eligibility criteria for *BRCA1* and *BRCA2* mutation testing based on family history and age of onset will identify only a small proportion of mutation carriers. © 2000 CancerResearch Campaign


*British Journal of Cancer* (2000) **83**(10), 1301–1308
© 2000 Cancer Research Campaign
doi: 10.1054/ bjoc.2000.1407, available online at http://www.idealibrary.com on IDEAL®

### Prevalence and penetrance of *BRCA1* and *BRCA2* mutations in a population-based series of breast cancer cases

**Anglian Breast Cancer Study Group**

See Appendix 2 for contributors

**Summary** Estimates of the contribution of *BRCA1* and *BRCA2* to breast cancer incidence in outbred populations have been based on studies that are either small or have selected for cases diagnosed at an early age. Only one of these has reported an estimate of the breast cancer risk associated with a mutation in these genes, and there is no published ovarian cancer risk estimate derived from a population-based case series. We screened a population-based series of breast cancer cases diagnosed before the age of 55 for mutations in *BRCA1* and *BRCA2*. Pedigree information from the mutation carriers was used to estimate penetrance and the proportion of familial risk of breast cancer due to *BRCA1* and *BRCA2*. We identified eight (0.7%) *BRCA1* and 16 (1.3%) *BRCA2* mutation carriers in 1220 breast cancer cases (actual sample size 1435 adjusted for 15% polymerase chain reaction failure rate). Mutation prevalence was substantially higher in cases diagnosed before 35 years-of-age and with increasing number of relatives affected with breast or ovarian cancer. However, most mutation carriers were diagnosed in the older age groups, and a minority reported a first-degree relative with breast cancer. Breast cancer penetrance by age 80 was estimated to be 48% (95% CI 7–82%) for *BRCA1* mutation carriers and 74% (7–94%) for *BRCA2* mutation carriers. Ovarian cancer penetrance for *BRCA1* and *BRCA2* combined was 22% (6–65%) by age 80. 17% of the familial risk of breast cancer was attributable to *BRCA1* and *BRCA2*. At birth, the estimated prevalence of *BRCA1* mutation carriers was 0.07% or 0.09% depending on the penetrance function used for the calculation. For *BRCA2*, the birth prevalence estimates were 0.14% and 0.22%. Mutations in the genes *BRCA1* and *BRCA2* are rare in the population and account for a small fraction of all breast cancer in the UK. They account for less than one fifth of the familial risk of breast cancer. Eligibility criteria for *BRCA1* and *BRCA2* mutation testing based on family history and age of onset will identify only a small proportion of mutation carriers. © 2000 Cancer Research Campaign

**Keywords** breast; ovarian; cancer; BRCA1; BRCA2; prevalence; penetrance

The breast-ovarian cancer susceptibility genes *BRCA1* and *BRCA2* were isolated in 1994 (Miki et al, 1994) and 1995 (Wooster et al, 1995) respectively. Since then, over thirty studies have investigated the frequency of mutations in these genes in high-risk breast and breast-ovarian cancer families (Gayther et al, 1998). Estimates of the proportion of families due to *BRCA1* and *BRCA2* have ranged from 6–79%. The probability of a mutation being identified increases with the number of ovarian cancers and breast cancers in the family, and with lower average age-at-diagnosis of breast cancer in the family (Couch et al, 1997; Ford et al, 1998). High-risk families have also been used to estimate the cumulative risks (penetrance) of breast and ovarian cancer in mutation carriers. For breast cancer, estimates of penetrance by age 70 in *BRCA1* and *BRCA2* mutation carriers have ranged from 50–85% (Easton et al, 1995; 1997; Ford et al, 1994). For ovarian cancer penetrance by age 70 has been estimated to be 62–66% for *BRCA1* (Ford et al, 1994; Easton et al, 1997; Antoniou et al, 1999), but about half this for *BRCA2* (Ford et al, 1998; Antoniou et al, 1999). Because these data are from high-risk families the results may not apply to all carriers of *BRCA1* or *BRCA2* mutations.

*Received 22 March 2000*
*Revised 22 June 2000*
*Accepted 26 June 2000*

*Correspondence to*: PDP Pharoah (Email: paul.pharoah@srl.cam.ac.uk)

Mutation analysis of *BRCA1* and *BRCA2* is laborious because of the large size of the genes and the diversity of mutations. However, several population isolates have a restricted number of founding mutations and so are more amenable to population-based studies of these genes. One per cent of the Ashkenazi Jewish population carry the mutation *BRCA1* 185delAG (Struewing et al, 1995) and 1.2% carry the *BRCA2* 6174delT mutation. These two mutations account for 20% and 8%, respectively, of early-onset breast cancer in this population (Neuhausen et al, 1996; Offit et al, 1996). In Iceland, a single mutation in *BRCA2* (999del5) is found in 24% of women diagnosed before age 40, with little contribution from *BRCA1* (Johannesdottir et al, 1996; Thorlacius et al, 1997). Penetrance estimates from population-based studies in these populations have generally been lower than those derived from the high-risk families (Ford et al, 1994; Easton et al, 1995; 1997; Antoniou et al, 1999). By the age of 70, carriers of the common Ashkenazi mutations were estimated to have a breast cancer risk of 56% and a 16% risk of ovarian cancer (Struewing et al, 1997) and carriers of the Icelandic mutation in *BRCA2* had a breast cancer risk of 38% (Thorlacius et al, 1998). These prevalence estimates (and perhaps also the penetrance estimates) cannot be extrapolated to a large outbred population such as the UK.

There have now been several estimates of the contribution of *BRCA1* and *BRCA2* to breast cancer incidence in different outbred populations. Two studies from the USA have tested patients under 35 for mutations in *BRCA1*: Langston et al (1996) found five (one of six mutations reported now not thought to be disease-causing (*BRCA1* 5396 + 60ins12)) mutation carriers out of 80 (6.3%) and Malone et al (1998) 12 out of 193 (6.2%). Hopper et al (1999) screened *BRCA1* in 91 Australian women diagnosed with breast cancer before the age of 40 and found three mutations (3.8%). The largest population-based study published to date, carried out in the UK, tested 619 cases diagnosed under 45 for mutations in *BRCA1* and *BRCA2*. *BRCA1* mutations were found in 2.6% of the cases and 2.3% of the cases carried mutations in *BRCA2* (Peto et al, 1999). The only study to include patients older than 44 found *BRCA1* mutations in three of 211 (1.4%) patients aged 20–74 (Newman et al, 1998). No penetrance estimates for mutations in these populations have been published.

We have screened a population-based series of 1435 cases diagnosed with breast cancer before the age of 55 for mutations in *BRCA1* and *BRCA2*. To our knowledge this is the largest study to screen the whole of the coding region of both *BRCA1* and *BRCA2* for mutations yet carried out, and the only population-based study of patients diagnosed over the age of 45. Pedigree information from the mutation carriers we identified was used to estimate penetrance and to estimate the proportion of familial relative risk of breast cancer due to *BRCA1* and *BRCA2*. We have also used these data to estimate the prevalence of *BRCA1* and *BRCA2* mutation carriers at birth.

### METHODS

### Case collection

The breast cancer case collection covered the region served by the Anglian Cancer Registry and all women diagnosed with breast cancer under the age of 55 between 1 January 1991 and 30 June 1996, who were alive at the start of the study on 1 July 1996, were eligible for inclusion. The study was approved by all the Local Research Ethics Committees in East Anglia. Patient details were obtained from the Anglian Cancer Registry, and the patients' general practitioners and consultants were contacted in order to obtain permission to contact the patients. Patient contact was delayed for at least 3 months after the initial diagnosis. Women taking part in the study were asked to provide a 20 ml blood sample and to complete a comprehensive epidemiological questionnaire. This included questions on family history, reproductive history (menarche, pregnancies, menopause), oral contraceptive pill and hormone replacement therapy use, past medical history and previous examination of the breast by mammography. The family history included details of all first-degree relatives to include date and place of birth, date of death, and any history of cancer. Details of any other relatives known by the case to have had cancer were also ascertained. Cancer diagnoses reported by the index cases were not independently verified. The Anglian Cancer Registry actively follows-up all notified cancer cases, and so we were able to ascertain second cancer diagnoses and vital status of the index cases at the end of the study.

### Mutation detection

Genomic DNA was extracted from blood using standard methods on an Extragen automated DNA extractor. The entire coding sequence and intron–exon boundaries of *BRCA1* and *BRCA2* was amplified by polymerase chain reaction (PCR) using 67 primer pairs. The double-stranded DNA fragments were screened for the presence of mismatched bases by multiplex heteroduplex analysis (MHA) with a mildly denaturing polyacrylamide gel matrix (10% polyacrylamide (99:1 bis-acryloyl piperazine), 10% ethylene glycol and 15% formamide) (Ganguly et al, 1993). Up to four fragments from the same patient, each differing in size by approximately 100 base pairs, were run in one lane of the gel. Electrophoresis was performed for 14 h at a constant 200 V, with gels cooled to 10°C, using the Protean II™ vertical slab gel apparatus. All gels were stained with silver nitrate according to standard protocols. Mutations were confirmed by direct sequencing of the PCR product in which a heteroduplex analysis variants had been found.

### Statistical analysis

The proportion of cases with mutations in different age groups and with different categories of family history were estimated directly. Ninety-five percent confidence intervals were calculated by standard methods.

#### *Population frequency of BRCA1 and BRCA2 mutations*

The frequency of *BRCA1* and *BRCA2* mutation carriers in the population was derived from the gene-specific penetrance and the proportions of cases due to *BRCA1* and *BRCA2* in different age-bands. Assuming that gene mutation carriers have mortality from causes other than breast cancer that are the same as the general population, the carrier frequency in the population, $p$, can be estimated by solving the equation:

$$\text{observed number of carriers} = p\Sigma n_i(f_i - f_{i-1})g_i/(f'_i - f'_{i-1})g'_i$$

Where $f_i$ and $f'_i$ denote breast cancer penetrance in mutation carriers and the general population respectively, at the end of the $i$th 5-year age interval, $g_i$ and $g'_i$ the probability of not developing ovarian cancer in mutation carriers and the general population, and $n_i$ the number of index cases in the $i$th age-group in our study. This calculation was repeated using our penetrance estimates and those reported by the Breast Cancer Linkage Consortium (BCLC) (Ford et al, 1998).

#### *Fraction of breast cancer familial relative risk due to BRCA1 and BRCA2*

Expected number of breast cancers occurring before age 85 among first-degree relatives of index cases were calculated from age- and period-specific incidence rates for England and Wales using the PERSON-YEARS program (Coleman et al, 1986). Individuals were followed from 1960 or from date of birth if born after 1960, and were censored at reported date of diagnosis of cancer, date of death, or date the family history questionnaire was completed. The number of breast cancers in excess of those expected in relatives of mutation carriers was compared to the excess for all relatives, to give the fraction of breast cancer familial relative risk due to *BRCA1* and *BRCA2*.

#### *Breast cancer penetrance estimation*

We used the pedigree information for the mutation carriers to estimate the penetrance of *BRCA1* and *BRCA2* using the program MENDEL. Since ascertainment was through a single affected index case, we maximized the conditional likelihood of the pedigree given the phenotypic and genotypic information of the index

 *© 2000 Cancer Research Campaign*

case, i.e. *L(pedigree|index) = L(pedigree)/L(index)*. Non-gene carriers were assumed to develop the disease at population incidence rates. We parameterized the model in terms of log relative risk for breast and ovarian cancer in mutation carriers compared to general population risks. The breast cancer relative risk was allowed to vary with age using three age-groups, 20–39, 40–59 and 60–79. Ovarian cancer relative risk was kept constant with age. Cumulative risks and associated 95% confidence intervals were calculated by the method given in Appendix 1.

### RESULTS

### *BRCA1* and *BRCA2* mutation prevalence

Of 2805 women diagnosed with breast cancer under the age of 55 between 1 January 1991 and 30 June 1996, 569 had died and 200 were thought by their general practitioner to be unfit to take part at the start of the study. Thus there were 2028 women eligible for inclusion in the study, of whom 1486 (73%) completed a questionnaire and 1435 (71%) provided a blood sample. The entire coding sequence and intron–exon boundaries of *BRCA1* and *BRCA2* were screened for mutations by MHA of 67 separate fragments following amplification of DNA by polymerase chain reaction (PCR). Not all samples successfully amplified for each fragment; 85% of a total of 96 145 PCR reactions were sufficiently strong to score on the MHA gels. We therefore adjusted the number of samples screened by a factor of 0.85 to give an effective sample size of 1220.

We detected eight *BRCA1* mutations and 16 *BRCA2* mutations that were predicted to encode a truncated protein (Table 1). Two of these were splice-site mutations, *BRCA1* 5312 + 2delT and *BRCA2* 295 + 3T>C, which we believe are likely to be disease-associated. The first of these affected the donor splice-site consensus sequence and was found in a patient aged 31 with a strong family history, and has been reported previously on the Breast Information Core database. The second is previously unreported, and though not affecting the consensus sequence was also found in a young patient with a positive family history. cDNA from this patient was available to confirm the effects of this mutation, which results in skipping of exon 2. The aberrant splicing results in loss of the start of the normal open-reading frame with a predicted cryptic open-reading frame start-site in exon 3, which is out of frame and predicted to result in a protein of only 20 amino acids.

Three cases were found to have an in-frame deletion. The *BRCA1* variant 1224delGAT (D369del) was found in a patient aged 53 at diagnosis who had no family history of breast or ovarian cancer and is reported on the BIC database. The deleted amino acid is conserved in murine Brca1 but does not lie within any known functional domain. 4369delAAG (E1382del) in *BRCA2*, has also been reported on the BIC database, and was found in two patients; one aged 45 with no family history, and the other a 50-year-old patient who had a paternal aunt diagnosed with breast cancer aged 39. Again, the deleted amino acid is conserved in murine Brca2 but does not lie within any known functional domain. As the functional significance of these variants is uncertain we have not included them in the analysis.

**Table 1** Details of cases with identified mutation in *BRCA1* or *BRCA2*

| Sample | Age | Mutation | | Family history of breast or ovarian cancer | | Family history of |
|---|---|---|---|---|---|---|
| | | | | Mother or sister[a] | Other relative[b] | other cancer[b] |
| **BRCA1** | | | | | | |
| R0913 | 32 | 2774delC | Frameshift | M Br (35) | – | – |
| R1524 | 45 | 2379delG | Frameshift | Nil | – | – |
| R2233 | 52 | 3596delAAAG | Frameshift | Nil | – | – |
| R1744 | 43 | 1436insC | Frameshift | Nil | – | FCo(75), PU St(75) |
| R1482 | 49 | 1619delTAAAT | Frameshift | Nil | MA Br(66) | – |
| R1767 | 44 | 3874delTGTC | Frameshift | S Br (46) | – | – |
| R1305 | 44 | 4158delAG | Frameshift | Nil | PGM Br(67) | – |
| R2038 | 31 | 4912 + 2delT | Splice site | Nil | MGA Br (27,34), MGA Br(?), MGA Br(?), M2C Ov(?) | MGGM St(57) |
| **BRCA2** | | | | | | |
| R1133 | 34 | 295 + 3A>G | Splice site | M Br(50) | – | – |
| R1125 | 46 | 253delC | Frameshift | Nil | PGM Br(80), PA Ov(57), PA Br(43, 57) | – |
| R1090 | 48 | 983delACAG | Frameshift | S Br(48) | PA Br(52), PA Br(44) | PU St(53), PU St(57) |
| R0495 | 46 | 1537delAAGA | Frameshift | Nil | – | F Pr(41), D Co(2) |
| R1725 | 32 | 1215insA | Frameshift | Nil | – | – |
| R0431 | 39 | 2023delTTACT | Frameshift | Nil | PGM Br (70) | – |
| R2119 | 41 | 3034delAAAC | Frameshift | M Br(72) | – | – |
| R1112 | 50 | 4538delA | Frameshift | Nil | – | – |
| R0739 | 54 | 4866delT | Frameshift | S Br(49), | MA Br(?) | F Co(68) |
| R1646 | 31 | 5849delTTAA | Frameshift | Nil | – | – |
| R0003 | 41 | 6503delTT | Frameshift | Nil | PA Br(38), PA Br(42) | – |
| R1486 | 36 | 6503delTT | Frameshift | Nil | PGM Br(46) | – |
| R1612 | 48 | 6719delTG | Frameshift | Nil | MA Ov(60) | – |
| R1846 | 48 | 9475delA | Frameshift | M Br(65), *S* Br (37, 52) | – | MGM St(?) |
| R0531 | 51 | 9303ins31 | Frameshift | M Br (74) | – | – |
| R1066 | 32 | 9303ins31 | Frameshift | Unknown | – | – |

[a] Br = author to supply; [b] MA = 000; PA = 000; MGM = 000; MGGM = 000; PGM = 000; MGA = 000; MZC = 000; D = 000; F = 000; PU = 000; St = 000; Co = 000

© 2000 Cancer Research Campaign 

**Table 2** Prevalence of *BRCA1* and *BRCA2* mutations in breast cancer cases by age

| Age group | Number | BRCA1 | | BRCA2 | | Total | |
|---|---|---|---|---|---|---|---|
| | | *n* | %[a] | *n* | %[a] | *n* | %[a] |
| <35 | 57 | 2 | 4.1 (0.5, 14.1) | 4 | 8.3 (2.3, 19.8) | 6 | 12.4 (4.7, 25.0) |
| 35–44 | 341 | 3 | 1.0 (0.2, 3.0) | 4 | 1.4 (0.4, 3.5) | 7 | 2.4 (1.0, 4.9) |
| 45–54 | 917 | 3 | 0.3 (0.1, 1.1) | 8 | 1.0 (0.4, 2.0) | 13 | 1.7 (0.9, 2.8) |
| Total | 1435 | 8 | 0.7 (0.3, 1.3) | 16 | 1.3 (0.8, 2.1) | 24 | 2.0 (1.3, 2.9) |

[a] Adjusted for 15% failure rate in DNA amplification by PCR for mutation analysis

**Table 3** Prevalence of *BRCA1* and *BRCA2* mutations in breast cancer cases by family history

| Family history of breast or ovarian cancers | Breast any age | | 1 breast < 60 years | | 1 breast < 50 years | |
|---|---|---|---|---|---|---|
| | *n* | Mutation (%[a]) | *n* | Mutation (%[a]) | *n* | Mutation (%[a]) |
| No more than one second-degree | 1124 | 11(1) | | | | |
| One first-degree | 136 | 5(4) | 111 | 5(5) | 84 | 2(3) |
| breast | 119 | 5(5) | 94 | 5(6) | 67 | 2(4) |
| ovary | 17 | 0 | 17 | 0 | 17 | 0 |
| Two first-or second-degree | 70 | 3(5) | 45 | 3(8) | 34 | 2(10) |
| Two first | 9 | 1(13) | 7 | 1(17) | 4 | 1(29) |
| One first | 33 | 1(4) | 22 | 1(5) | 18 | 1(7) |
| One or two first | 42 | 2(6) | 29 | 2(8) | 22 | 2(11) |
| Two second | 28 | 1(4) | 16 | 1(7) | 11 | 1(11) |
| Three first- or second-degree | 6 | 2(39) | 6 | 2(39) | 4 | 2(59) |
| Four + relatives | 12 | 1(10) | 11 | 1(11) | 9 | 1(13) |
| One first- or second-degree ovary | 44 | 2(6) | | | | |

[a] Adjusted for 15% PCR failure rate

We identified 22 missense variants that occurred in up to four cases, accounting for 31 cases in total. Fifteen of these have been previously reported on the BIC database (at http://www.nhgri.nih.gov/Intramural_research/Lab_transfer/Bic/index.html). The remaining seven are novel variants. Although it is possible that some of these are disease-causing, there are no adequate methods to assess their effect on protein function, and so we have not classified any of them as disease-associated.

The overall mutation prevalence was low, 0.7% for *BRCA1* and 1.3% for *BRCA2*, but was significantly higher in cases diagnosed under 35 years-of-age ($P < 0.001$) (Table 2). There was no apparent difference in the age distribution of cases due to *BRCA1* and *BRCA2* in our series. These proportions underestimate the true contribution of *BRCA1* and *BRCA2* to breast cancer incidence because a substantial fraction of mutations, for example large deletions, are missed by screening just the coding sequence. By comparing the frequency of known mutations in families linked to *BRCA1*, the BCLC estimated the sensitivity of MHA and similar techniques to be 63% (Ford et al, 1998). On this basis, the overall proportion of breast cancer below age 55 due to *BRCA1* and *BRCA2* is estimated to be 1.2% and 2.1% respectively.

One of the patients who was found to have a mutation did not complete her family history questionnaire. Of the remaining 23 mutation carriers, seven reported no family history of breast or ovarian cancer. Two of the eight *BRCA1* and six of 16 *BRCA2* mutation carriers had a mother or sister affected with breast cancer, and two *BRCA1* and five *BRCA2* mutation carriers reported at least one second-degree relative with breast or ovarian cancer. Only three mutation carriers reported a family history of ovarian cancer. Table 3 shows the number of women in different family history categories that were found to have mutations.

One hundred and seventy seven breast cancers were reported in female first-degree relatives of the index cases, compared to 105.8 expected from age- and period-specific incidence rates. Eight mothers or sisters of mutation carriers developed breast cancer compared to 1.47 expected. If these data are adjusted for the effective sample size of 85% of the total, and for a mutation detection sensitivity of 63%, 17% of the excess risk to relatives is attributable to *BRCA1* and *BRCA2* mutations.

We have estimated the proportion of individuals in the general population who are carriers of mutations in *BRCA1* or *BRCA2* at birth. Assuming a mutation detection sensitivity of 63%, the estimated prevalences of *BRCA1* and *BRCA2* carriers at birth are 0.09% and 0.22% respectively, using our penetrance estimates (see below), and 0.07% and 0.14% if the BCLC penetrance function is used.

### *Breast cancer risks in* BRCA1 *and* BRCA2 *mutation carriers*

The maximum likelihood estimates of the parameters for the breast and ovarian cancer penetrance models are given in Table 4. The relative risk of breast cancer for *BRCA1* mutation carriers decreased with age, but for *BRCA2* mutation carriers the relative risk for the three age-groups varied little. The breast and ovarian cancer cumulative risk (penetrance) estimates based on the estimated age-specific relative risks are presented in Table 5. The risk associated with mutations in *BRCA1* was slightly higher than for *BRCA2* up to age 60, and slightly lower thereafter, but these estimates are based on a small number of families, and there is a substantial overlap in the associated confidence intervals.

No first-degree relatives of the index cases were reported as having ovarian cancer, but two of the index cases (one *BRCA1* and one *BRCA2*) were diagnosed with ovarian cancer after they had

*© 2000 Cancer Research Campaign*

**Table 4** Penetrance model parameter estimates

| Mutation | Model loglikelihood | Parameter[a] | Breast cancer | | | Ovarian cancer |
|---|---|---|---|---|---|---|
| | | | 20–39 | 40–59 | 50–79 | 20–79 |
| *BRCA1/2* | –66.3 | Relative risk | 26.4 | 11.8 | 17.6 | 15.9 |
| | | Ln (RR) | 3.27 | 2.47 | 2.87 | 2.77 |
| | | SE Ln (RR) | 0.73 | 0.60 | 0.76 | 0.75 |
| *BRCA1* | –17.1 | Relative risk | 58.4 | 14.7 | 1.00 | 41.8 |
| | | Ln (RR) | 4.07 | 2.67 | 0.00 | 3.73 |
| | | SE Ln (RR) | 1.10 | 1.33 | 0.00 | 1.25 |
| *BRCA2* | –47.8 | Relative risk | 17.2 | 11.2 | 22.5 | 9.9 |
| | | Ln (RR) | 2.84 | 2.42 | 3.11 | 2.29 |
| | | SE Ln (RR) | 1.02 | 0.68 | 0.75 | 1.03 |

[a] < n = ; SE = (author to supply)

**Table 5** Cumulative breast and ovarian cancer risks (%) in *BRCA1* and *BRCA2* mutation carriers

| Age | Gen population[a] | BRCA1[b] | BRCA2[b] | All[b] |
|---|---|---|---|---|
| *Breast cancer* | | | | |
| 40 | 0.4 | 20 (0–50) | 6 (0–17) | 10 (0–21) |
| 50 | 1.5 | 32 (2–62) | 18 (2–32) | 21 (5–34) |
| 60 | 3.1 | 46 (3–82) | 31 (3–53) | 34 (5–55) |
| 70 | 5.0 | 47 (5–82) | 56 (5–80) | 54 (14–76) |
| 80 | 7.1 | 48 (7–82) | 74 (7–94) | 69 (11–90) |
| *Ovarian cancer* | | | | |
| 40 | 0.1 | 3 (0–30) | 1 (0–5) | 1 (0–5) |
| 50 | 0.3 | 11 (1–74) | 3 (0–19) | 4 (1–18) |
| 60 | 0.7 | 24 (2–96) | 6 (1–39) | 10 (2–37) |
| 70 | 1.0 | 36 (4–99) | 10 (1–55) | 16 (4–51) |
| 80 | 1.5 | 47 (5–100) | 14 (2–68) | 22 (6–65) |

[a] Based on 1983–87 incidence for England and Wales; [b] 95% confidence interval in brackets been diagnosed with breast cancer. The estimated cumulative risk of ovarian cancer by age 70 for *BRCA1* and *BRCA2* combined was 16% (4–52%).

### DISCUSSION

Our mutation prevalence estimates show the expected decline with increasing age, and are broadly in line with estimates from other population-based studies. We found that 4.1% of cases diagnosed under 35 years-of-age carried a mutation in *BRCA1*. This is comparable with other published estimates from population-based studies in this age-group – 6.3% (Langston et al, 1996), 6.2% (Malone et al, 1998) and 3.5% (Peto et al, 1999) cases under 36. Our estimate of prevalence of *BRCA2* mutations in the same age-group was somewhat higher than those previously reported: 8.3% compared with 2.4% (Peto et al, 1999) and 2.2% (Krainer et al, 1997), but there is a substantial overlap between the confidence intervals of these estimates. In cases aged 35–44 we found 1.0% with *BRCA1* mutations and 1.0% with *BRCA2* mutations compared with 1.9% and 2.2% reported by Peto et al (1999). We believe that our estimate of mutation prevalence in older cases (aged 45–54) is the only population-based series of breast cancer cases that has been screened for mutations in both *BRCA1* and *BRCA2*.

The observed estimates of mutation prevalences are likely to be underestimates. Firstly, although we have not classified any of the rare missense variants and in-frame deletions as disease-causing, this may be incorrect for some of them. In addition, the sensitivity of the mutation detection technique is incomplete and large genomic deletions, mutations in the promoter region and an unknown proportion of single-base substitutions will be missed. *BRCA1* spans approximately 80 kb of genomic DNA in a region containing an unusually high density of Alu repeats, which may make it relatively unstable and prone to deletions and rearrangements (Smith et al, 1996). Indeed, 36% of mutations in Dutch families are due to three different, large genomic deletions in *BRCA1* (Petrij Bosch et al, 1997), and duplication of exon 13 has been reported in a BCLC family linked to *BRCA1*. A large deletion in *BRCA2* has also been reported in a Swedish family (Nordling et al, 1998). Unfortunately, unless the specific mutation is known, testing for large genomic alterations is laborious and requires substantial quantities of DNA, and is not feasible in the context of a large population-based study. There are, however, estimates of the sensitivity of techniques such as MHA, and using families clearly linked to either gene, the sensitivity of standard mutation detection techniques has been shown to be around 63%. If our data are adjusted for a mutation sensitivity of 63%, the overall *BRCA1* mutation prevalence becomes 1.3% and for *BRCA2*, 2.1%.

The mutation prevalence estimates may also be biased by preferential survival between cases with mutations and those without. Several studies have compared survival of *BRCA1*-associated breast cancer to that of *BRCA1*-negative cases with conflicting results (Ansquer et al, 1998; Verhoog et al, 1998; Watson et al, 1998). However, the pathological features of *BRCA1*-associated tumours would predict a poorer survival in this group of patients. If the survival of *BRCA1*-associated tumours was half that of

*© 2000 Cancer Research Campaign* 

*BRCA1*-negative cases, the estimated *BRCA1*-mutation prevalence would increase by a factor of 1.3. There are no data on survival of *BRCA2*-associated breast cancer, but these tumours tend to have a histopathology comparable with that of control (Breast Cancer Linkage Consortium, 1997).

As expected, the proportion of women with a mutation varied according to family history, the proportion increasing with increasing number of affected relatives. Several studies have shown that the probability of finding a mutation in *BRCA1* or *BRCA2* in breast cancer families is substantially greater if there is at least one relative affected with ovarian cancer. Although none of 24 cases with a single first-degree relative affected with ovarian cancer were found to be mutation carriers, a mutation was found in three of 55 cases that reported ovarian cancer in at least one relative.

These findings have important implications for determining policy for *BRCA1* and *BRCA2* mutation testing. Eligibility criteria for testing should take into account their sensitivity and positive predictive value. In this context, the sensitivity is the proportion of the cases who are mutation carriers who are identified by the criteria, and the positive predictive value is the proportion of those who fulfil the criteria who are found to be mutation carriers. Any criteria based on family history alone or in combination with age of onset will have a limited sensitivity because a significant proportion of the total number of mutation carriers identified occurs in older cases with little or no family history. For example, if all women diagnosed with breast cancer under 35 years-of-age and those with at least two affected relatives on the same side of the family, one of whom was diagnosed aged less than 50, were eligible for testing, a mutation in *BRCA1* or *BRCA2* would be found in approximately 10% (the positive predictive value). These criteria would have identified 10 out of the 24 mutation carriers identified in the ABC study, a sensitivity of 42%. More stringent criteria will improve the positive predictive value, but at the expense of sensitivity.

We found that only 17% of the breast cancer familial relative risk can be accounted for by *BRCA1* and *BRCA2*, which suggests that there may be other breast cancer susceptibility genes. However, because the proportion of cases with a mutation increases as the familial clustering increases, these putative genes are likely to have a lower penetrance than *BRCA1* and *BRCA2*. This hypothesis is consistent with the findings of Peto et al (1999) and with a recent analysis of the BCLC families, which found that only one-third of site-specific breast cancer families with only four or five affected members were due to *BRCA1* or *BRCA2* (Ford et al, 1998). Our estimates of the prevalence of the two genes in the general population are similar to those published elsewhere (Ford et al, 1995; Peto et al, 1999).

Although the cumulative breast cancer risk by age 70 was higher for *BRCA2* than for *BRCA1*, these were not statistically significantly different. Like other penetrance estimates derived from population-based studies (Struewing et al, 1997; Thorlacius et al, 1998; Hopper et al, 1999), breast cancer risks were somewhat lower than those derived from high-risk families (Ford et al, 1998). Our estimate of ovarian cancer penetrance was also lower than the estimates derived from high-risk families, and identical to the only published estimate of ovarian cancer penetrance derived from a population-based study of breast cancer (Struewing et al, 1997). Other estimates for *BRCA1* ovarian cancer penetrance by age 70 have ranged from 53% using data from ovarian cancer families (Antoniou et al, 1999) to 63% based on the BCLC families (Easton et al, 1995). Antoniou et al (1999) also estimated the ovarian cancer penetrance by age 70 to be 68% using data from a population-based study of ovarian cancer. The two published estimates of *BRCA2* ovarian cancer penetrance have been lower: 27% using BCLC families (Ford et al, 1998) and 31% using ovarian cancer families. The reasons for the differences in ovarian cancer risks derived from the population-based studies and the high-risk families are not clear. Site-specific differences in breast and ovarian cancer risk (allelic heterogeneity) have been described for mutations in both *BRCA1* (Gayther et al, 1995) and *BRCA2* (Gayther et al, 1997), but these effects do not seem large enough to explain the observed risk differences, and modifying genes segregating in the high-risk families may also be important.

### ACKNOWLEDGEMENTS

We thank Dr Tom Davies and the Anglian Cancer Registry for access to their data, all the general practitioners in East Anglia for permission to contact their patients and for taking the blood samples, and breast surgeons, oncologists and breast nurses in East Anglia for helping make this study possible. S McBride, P Harrington, P Russell, C Healey and N Foster assisted with DNA preparation. The ABC Study was funded by NHS R&D grants RG22086 and NCP/B15. PDPP (see Appendix 2) is a Cancer Research Campaign (CRC) Senior Clinical Research Fellow, BAJP is a Gibb Fellow of the CRC.

### REFERENCES

Ansquer Y, Gautier C, Fourquet A, Asselain B and Stoppa-Lyonnet D (1998) Survival in early-onset *BRCA1* breast cancer patients. *Lancet* **352**: 541

Antoniou AC, Gayther SA, Stratton JF, Ponder BAJ and Easton DF (2000) Risk models for familial breast and ovarian cancer. *Genet Epidemiol* **18**: 173–190

Breast Cancer Linkage Consortium (1997) Pathology of familial breast cancer: differences between breast cancers in carriers of *BRCA1* or *BRCA2* mutations and sporadic cases. *Lancet* **349**: 1505–1510

Coleman M, Douglas A, Hermon C and Peto J (1986) Cohort study analysis with a FORTRAN computer program. *Int J Epidemiol* **15**: 134–137

Couch FJ, Deshano ML, Blackwood MA, Calzone K, Stopfer J, Campeau L, Ganguly A, Rebbeck T and Weber BL (1997) *BRCA1* mutations in women attending clinics that evaluate the risk of breast cancer. *N Engl J Med* **336**: 1409–1415

Easton DF, Ford D, Bishop DT and Breast Cancer Linkage Consortium (1995) Breast and ovarian cancer incidence in *BRCA1*-mutation carriers. *Am J Hum Genet* **56**: 265–271

Easton DF, Steele L, Fields P, Ormiston W, Averill D, Daly PA, McManus R, Neuhausen SL, Ford D, Wooster R, Cannon Albright LA, Stratton MR and Goldgar DE (1997) Cancer risks in two large breast cancer families linked to *BRCA2* on chromosome 13q 12–13. *Am J Hum Genet* **61**: 120–128.

Ford D, Easton DF, Bishop DT, Narod SA, Goldgar DE and Breast Cancer Linkage Consortium (1994) Risks of cancer in *BRCA1* mutation carriers. *Lancet* **343**: 692–695.

Ford D, Easton DF and Peto J (1995) Estimates of the gene frequency of *BRCA1* and its contribution to breast and ovarian cancer incidence. *Am J Hum Genet* **57**: 1457–1462.

Ford D, Easton DF, Stratton M, Narod S, Goldgar D, Devilee P, Bishop DT, Weber B, Lenoir G, Chang-Claude J, Sobol H, Teare MD, Struewing J, Arason A, Scherneck S, Peto J, Rebbeck TR, Tonin P, Neuhausen S, Barkadottir R, Eyfjord J, Lynch H, Ponder BAJ, Gayhter SA, Birch JM, Lindblom A, Stoppa-Lyonnet D, Bignon Y, Borg A, Hamann U, Haites N, Scott RJ, Maugard CM, Vasen H, Seitz S, Cannon-Albright LA, Schofield A, Hedman MZ and Breast Cancer Linkage Consortium (1998) Genetic heterogeneity and penetrance analysis of the *BRCA1* and *BRCA2* genes in breast cancer families. *Am J Hum Genet* **62**: 676–689

Ganguly A, Rock MJ and Prockop DJ (1993) Conformation-sensitive gel electrophoresis for rapid detection of single-base difference in double stranded

 *© 2000 Cancer Research Campaign*

PCR products and DNA fragments: evidence for solvent-induced bends in DNA heteroduplexes. *Proc Natl Acad Sci USA* **90**: 10325–10329

Gayther SA, Mangion J, Russell P, Seal S, Barfoot R, Ponder BA, Stratton MR and Easton D (1997) Variation of risks of breast and ovarian cancer associated with different germline mutations of the *BRCA2* gene. *Nat Genet* **15**: 103–105

Gayther SA, Pharoah PDP and Ponder BAJ (1998) The genetics of inherited breast cancer. *J Mamm Gland Biol Neoplasia* **3**: 365–376

Gayther SA, Warren W, Mazoyer S, Russell PA, Harrington PA, Chiano M, Seal S, Hamoudi R, van Rensburg EJ, Dunning AM, Love R, Evans G, Easton D, Clayton C, Stratton MR and Ponder BAJ (1995) Germline mutations of the *BRCA1* gene in breast and ovarian cancer families provide evidence for a genotype–phenotype correlation. *Nat Genet* **11**: 428–433.

Hopper JL, Southey MC, Dite GS, Jolley DJ, Giles GG, McCredie MR, Easton DF, Venter DJ and Australian Breast Cancer Family Study (1999) Population-based estimate of the average age-specific cumulative risk of breast cancer for a defined set of protein-truncating mutations in *BRCA1* and *BRCA2*. *Cancer Epidemiol Biomarkers Prev* **8**: 741–747

Johannesdottir G, Gudmundsson J, Bergthorsson JT, Arason A, Agnarsson BA, Eiriksdottir G, Johannsson OT, Borg A, Ingvarsson S, Easton DF, Egilsson V and Barkardottir RB (1996) High prevalence of the 999del5 mutation in Icelandic breast and ovarian cancer patients. *Cancer Res* **56**: 3663–3665

Krainer M, Silva Arrieta S, Fitzgerald MG, Shimada A, Ishioka C, Kanamaru R, MacDonald DJ, Unsal H, Finkelstein DM, Bowcock A, Isselbacher KJ and Haber DA (1997) Differential contributions of *BRCA1* and *BRCA2* to early-onset breast cancer. *N Engl J Med* **336**: 1416–1421

Langston A, Malone KE, Thompson JD, Daling JR and Ostrander EA (1996) *BRCA1* mutations in a population-based sample of young women with breast cancer. *N Engl J Med* **334**: 137–142

Malone KE, Daling JR, Thompson JD, O'Brien CA, Francisco LV and Ostrander EA (1998) *BRCA1* mutations and breast cancer in the general population. *JAMA* **279**: 922–929

Miki Y, Swensen J, Shattuck-Eidens D, Futreal PA, Harsham K, Tavtigian S, Liu Q, Cochran C, Bennett LM, Ding W, Bell R, Rosenthal J, Hussey C, Tran T, McClure M, Frye C, Hattier T, Phelps R, Haugenstrano A, Katcher H, Yakumo K, Gholami Z, Shaffer D, Stone S, Bayer S, Wray C, Bogden R, Dayananth P, Ward J, Tonin P, Narod S, Bristow PK, Norris FH, Helvering L, Morrison P, Rosteck P, Lai M, Barrett JC, Lewis C, Neuhausen S, Cannon-Albright L, Goldgar D, Wiseman R, Kamb A and Skolnick MH (1994) A strong candidate for the 17-linked breast and ovarian cancer susceptibility gene *BRCA1*. *Science* **266**: 66–71

Neuhausen S, Gilewski T, Norton L, Tran T, McGuire P, Swensen J, Hampel H, Borgen P, Brown K, Skolnick M, Shattuck Eidens D, Jhanwar S, Goldgar D and Offit K (1996) Recurrent *BRCA2* 6174delT mutations in Ashkenazi Jewish women affected by breast cancer. *Nat Genet* **13**: 126–128

Newman B, Mu H, Butler LM, Millikan RC, Moorman PG and King M-C (1998) Frequency of breast cancer attributable to *BRCA1* in a population-based series of American women. *JAMA* **279**: 915–921

Nordling M, Karlsson P, Wahlstrom J, Engwall Y, Wallgren A and Martinsson T (1998) A large deletion disrupts the exon 3 transcription activation domain of the *BRCA2* gene in a breast/ovarian cancer family. *Cancer Res* **58**: 1372–1375

Offit K, Gilewski T, McGuire P, Schluger A, Hampel H, Brown K, Swensen J, Neuhausen S, Skolnick M, Norton L and Goldgar D (1996) Germline *BRCA1* 185delAG mutations in Jewish women with breast cancer. *Lancet* **347**: 1643–1645

Peto J, Collins N, Barfoot R, Seal S, Warren W, Rahman N, Easton DF, Evans C, Deacon J and Stratton MR (1999) Prevalence of *BRCA1* and *BRCA2* gene mutations in patients with early-onset breast cancer. *J Natl Cancer Inst* **91**: 943–949

Petrij Bosch A, Peelen T, van Vliet M, van Eijk R, Olmer R, Drusedau M, Hogervorst FB, Hageman S, Arts PJ, Ligtenberg MJ, Meijers Heijboer H, Klijn JG, Vasen HF, Cornelisse CJ, van't Veer LJ, Bakker E, van Ommen GJ and Devilee P (1997) *BRCA1* genomic deletions are major founder mutations in Dutch breast cancer patients. *Nat Genet* **17**: 341–345

Smith TM, Lee MK, Szabo CI, Jerome N, McEuen M, Taylor M, Hood L and King M-C (1996) Complete genomic sequence and analysis of 117kb of human DNA containing the gene *BRCA1*. *Genome Res* **6**: 1029–1049

Struewing JP, Abeliovich D, Peretz T, Avishai N, Kaback MM, Collins FS and Brody LC (1995) The carrier frequency of the *BRCA1* 185delAG mutation is approximately 1 percent in Ashkenazi Jewish individuals. *Nat Genet* **11**: 198–200

Struewing JP, Hartge P, Wacholder S, Baker SM, Berlin M, McAdams M, Timmerman MM, Brody LC and Tucker MA (1997) The risk of cancer associated with specific mutations of *BRCA1* and *BRCA2* among Ashkenazi Jews. *N Engl J Med* **336**: 1401–1408

Thorlacius S, Sigurdsson S, Bjarnadottir H, Olafsdottir G, Jonasson JG, Tryggvadottir L, Tulinius H and Eyfjord JE (1997) Study of a single *BRCA2* mutation with high carrier frequency in a small population. *Am J Hum Genet* **60**: 1079–1084

Thorlacius S, Struewing JP, Hartge P, Olafsdottir GH, Sigvaldason H, Tryggvadottir L, Wacholder S, Tulinius H and Eyfjord JE (1998) Population-based study of risk of breast cancer in carriers of *BRCA2* mutation. *Lancet* **352**: 1337–1339

Verhoog LC, Brekelmans CTM, Seynaeve C, van den Bosch LMC, Dahmen G, van Geel AN, Tilanus-Linthorst MMA, Bartels CCM, Wagner A, van den Ouweland A, Devilee P, Meijers-Heijboer EJ and Klijn JGM (1998) Survival and tumour characteristics of breast-cancer patients with germline mutations of *BRCA1*. *Lancet* **351**: 316–321

Watson P, Marcus JN and Lynch HT (1998) Prognosis of *BRCA1* hereditary breast cancer. *Lancet* **351**: 304–305

Wooster R, Bignell G, Lancaster J, Swift S, Seal S, Mangion J, Collins N, Gregory S, Gumbs C, Micklem G, Barfoot R, Hamoudi R, Patel S, Rice C, Biggs P, Hashim Y, Smith A, Connor F, Arason A, Gudmundsson J, Ficenec D, Kelsell D, Ford D, Tonin P, Bishop DT, Spurr NK, Ponder BAJ, Eeles R, Peto J, Devilee P, Cornelisse C, Lynch H, Narod S, Lenoir G, Egilsson V, Barkadottir RB, Easton DF, Bentley DR, Futreal PA, Ashworth A and Stratton MR (1995) Identification of the breast cancer susceptibility gene *BRCA2*. *Nature* **378**: 789–792

*© 2000 Cancer Research Campaign* 

### APPENDIX 1

Cumulative risk of penetrance and 95% confidence intervals (95% CI) were calculated from the cumulative incidence $\Lambda(t)$, where:

$$\Lambda(t) = \sum_{k=1}^{n} i_k t_k \exp(\beta_k)$$

$$\operatorname{var}\Lambda(t) = \sum_{k=1}^{n} i^2_k t^2_k \operatorname{var}(\beta_k)\exp(2\beta_k) + 2\sum_{\substack{j<k \\ k=1}}^{n} i_k i_j T_k T_j [\operatorname{var}(\beta_k)\operatorname{var}(\beta_j)]^{1/2} \exp(\beta_k)\exp(\beta_j)\operatorname{corr}(\beta_k, \beta_j)$$

where $i_k$ = incidence in the $k^{th}$ age-band of length $T_k$, and $\beta_k = \ln(RR)$ in $k^{th}$ age-band.

The cumulative risk F(t) is given by:

$$F(t) = 1 - \exp(-\Lambda(t))$$

with 95% CI = $1 - \exp(-\Lambda(t) \pm 1.96\sqrt{\operatorname{var}\Lambda(t)}$

### APPENDIX 2

### The Anglian Breast Cancer (ABC) Study Group

Principal investigators: Prof BAJ Ponder[1], Prof NE Day[2], Dr DF Easton[3] and Dr PDP Pharoah[1]
Writing committee: PDP Pharoah[1], DF Easton[3] and BAJ Ponder[1]
Study administration and co-ordination: JM Lipscombe[1]
Research Nurse: K Redman[3]
Statistical analysis: PDP Pharoah[1], DF Easton[3] and A Antoniou[3]
Laboratory analysis: V Basham[1], J Gregory[1] and PDP Pharoah[1]
Technical advisors: Dr S Gayther[1] and Dr A Dunning[1]
[1]Cancer Research Campaign Human Cancer Genetics Research Group, Department of Oncology; [2]Department of Public Health and General Practice; [3]Cancer Research Campaign Genetic Epidemiology Unit, Department of Public Health and General Practice, University of Cambridge, Strangeways Research Laboratories, Worts Causeway, Cambridge CB1 8RN, UK.

*© 2000 Cancer Research Campaign*
